# Measuring Assistive Technology Outcomes via AI-Based Kinematic Modeling of Individualized Routine Learning in Elite Boccia Athletes with Severe Cerebral Palsy: A Longitudinal Case Series

**DOI:** 10.3390/bioengineering13030261

**Published:** 2026-02-25

**Authors:** Se-Won Park, Young-Kyun Ha

**Affiliations:** 1 Department of Physical Education, Korea National University of Education, Cheongju 28173, Republic of Korea; parksewon@knue.ac.kr; 2 Next Generation Business Division, Airpass Co., Ltd., 15th Floor of D-dong, Hanam Techno Valley U1 Center, 947 Hanam-daero, Hanam-si 12982, Gyeonggi-do, Republic of Korea

**Keywords:** assistive technology, accessibility, artificial intelligence, Bidirectional LSTM, kinematic analysis, markerless motion capture, motor learning stages, Boccia, cerebral palsy

## Abstract

Objectives: This longitudinal single-case series evaluated an AI-based routine-learning system as assistive technology (AT) for elite Boccia athletes with severe Cerebral Palsy (CP). The study aimed to provide an innovative outcome measurement approach for individualized monitoring by integrating performance scores and longitudinal kinematic variability indicators. Methods: Three national-level players performed 694 throws over eight weeks. To ensure technical credibility, trials were rated through a consensus-based assessment by a panel of two experts, serving as ground truth for AI modeling. The system utilized a Bidirectional Long Short-Term Memory (Bi-LSTM) architecture to extract 29 kinematic features and perform regression-based scoring, providing real-time augmented feedback. Results: High-baseline tasks maintained stable scores (7–9), while intermediate tasks showed significant score increases, reflecting motor learning transitions. The model achieved a Mean Squared Error of 1.14 and a Mean Absolute Error of 1.13, demonstrating high alignment with expert standards. Training demonstrated stable convergence, with loss reducing from 7.45 to 1.19. Notably, for the most severely impaired athlete, the AI system detected a 4.69% reduction in kinematic variability despite stagnant performance scores. This provides empirical evidence of movement stabilization within the cognitive stage that traditional observation might overlook. Conclusions: The Bi-LSTM system enabled accurate tracking of performance and motor variability, revealing distinct learning curves based on task difficulty. These findings demonstrate the feasibility of AI-enabled motion analysis as an AT for outcome measurement, supporting data-driven coaching where conventional evaluation is constrained by the rarity and severity of disabilities.

## 1. Introduction

Boccia is an official Paralympic sport designed for individuals with severe cerebral palsy (CP) and other severe motor impairments, requiring highly precise throwing actions based on coordinated control of the upper limbs and trunk [1,2]. According to the Boccia International Sports Federation (BISFed), Boccia is defined as an adaptive sport that allows participation by athletes with severe motor disorders such as quadriplegia, dystonia, and trunk instability [3]. In school settings, Boccia provides an important educational resource for physical education classes and after-school sport programs, as it offers a competitive sport option even for students with markedly limited upper-limb function. Beyond participation itself, Boccia also provides a practical context in which accessibility-oriented support and individualized instruction can be operationalized through objective monitoring and feedback. Such inclusive participation aligns with the United Nations’ Sustainable Development Goals (SDGs), specifically by fostering equitable educational opportunities (SDG 4) and reducing social inequalities (SDG 10).

However, Boccia performance varies substantially depending on the pattern of impairment and functional characteristics (e.g., muscle tone patterns, upper-limb coordination, trunk stability) [4], and the highly individualized nature of technical execution makes instruction and assessment challenging. Even at the international level, coaches often rely on observational assessments and video review, and the development of performance assessment tools grounded in sport-specific demands has lagged behind. This gap threatens the sustainability of individualized instruction because practitioners lack objective data to document learning consistency and meaningful change over time in real practice contexts. From an assistive technology (AT) and accessibility perspective, this limitation also constrains outcome and impact measurement, as longitudinal improvement is difficult to substantiate without reliable indicators. The Five Target Test (FTT) proposed by González et al. [5] and the precision assessment protocols developed by Oliveira et al. [6,7] are early attempts to address these limitations and underscore the need for sport-specific evaluation. In addition, Roldan et al. [4] and Reina et al. [8] emphasize that impairment-related constraints—particularly spasticity and reduced trunk control—can meaningfully shape throwing mechanics and accuracy, reinforcing the need for individualized instruction aligned with functional capacity. As the effects of postural tilt and trunk stability on throwing accuracy in players with CP have been quantified [4,9], the importance of comprehensive analysis of postural control before, during, and after the throw has become increasingly evident.

Existing Boccia research has primarily focused on the biomechanics of the throwing motion [1], performance differences according to physical function [3,9], impairment-related constraints on kinematics and throwing accuracy under task demands (e.g., distance constraints) [8]. More recently, efforts to quantify Boccia performance have expanded toward field-relevant indicators that can function as measurable outcomes, which is increasingly important for establishing objective outcome measurement in disability sport contexts. Fong et al. [10] examined performance decrements in target hitting rate and maximum ball speed associated with muscle fatigue during a simulated prolonged Boccia game, while Tsai et al. [11] analyzed the effects of seat tilt adjustments on throwing movement characteristics and postural stability during Boccia ball throwing in children with CP, emphasizing the importance of trunk and seating configuration. The growing emphasis on quantitative analysis reflects increasing evidence that throwing velocity and accuracy are associated in Boccia players with CP [8], thereby amplifying the need to precisely measure fine-grained kinematic variables—such as swing kinematics and release-related parameters (e.g., release timing and launch angle) [12]. Roldan et al. [2,4] further contributed by developing reliable instruments to quantify hand, arm, and trunk function, thereby advancing the structural understanding of key performance components in Boccia. In a similar vein, Kataoka et al. [13] reported that shoulder range of motion and upper-limb strength are closely associated with throwing distance and accuracy, and Peña-González et al. [5] proposed the FTT as a standardized protocol for precision assessment.

Intervention studies on technical training are also growing in number. Yahagi et al. [14] demonstrated that rolling practice designed to reflect the specific demands of Boccia throwing is more effective than general upper-limb exercises for improving performance, and Sufitriyono et al. [15] showed that hand–eye coordination training can improve accuracy. These findings regarding repetitive skill training align with research on accuracy in closed, self-paced motor tasks that emphasizes the importance of pre-performance routines [16]. In the motor learning of children with CP, the effects of repetitive practice on motor variability have been highlighted [17], and the pattern of motor variability observed over the course of practice has been discussed as a functionally meaningful marker of motor learning and adaptability [18]. Furthermore, Vasconcelos et al. [19] used dual-energy X-ray absorptiometry (DXA) to analyze body composition and bone mineral density in world-class Boccia players, emphasizing the importance of long-term health and fitness factors, whereas Barak et al. [20] reported positive psychosocial effects of Boccia participation, and Koper et al. [21] examined the association between pre-competition psychological states and competitive results in disabled Boccia athletes.

A bibliometric analysis by Ferreira et al. [3], which systematically examined the expansion of Boccia research, showed that although the volume of research has increased over the past decade, studies remain concentrated on techniques, training, psychology, and classification. Crucially, there remains a paucity of studies utilizing field-based, longitudinal routine-learning data from real training or competition contexts. This also implies that evidence is limited regarding how accessibility-oriented support and AT-enabled approaches can incorporate objective outcome measurement to document meaningful longitudinal change in severe disability contexts. This underscores that the present study—an AI-based movement analysis focusing on time-series changes in routine learning among Boccia players with severe CP—addresses a specific international research gap regarding how repetitive practice reshapes performance and motor variability in this population.

With the advancement of artificial intelligence (AI) and sensor-based analytic technologies, studies have emerged that evaluate ramp angle, applied force, and movement patterns to enhance performance [22], analyze throwing-related arm gestures and movement patterns using wearable inertial sensors (e.g., wrist acceleration/rotation and applied force during ball throwing) [23], and employ virtual coaches through Boccia learning simulators [24]. These studies broaden the possibilities for quantitative analysis of Boccia technique; however, empirical applications involving students with disabilities remain scarce. In addition, when framed as an AT-enabled strategy, AI may improve accessibility to objective feedback and outcome monitoring in settings where expert coaching and repeated assessment are difficult to sustain. In upper-limb rehabilitation for children with CP, robot-assisted therapy has been shown to provide personalized interventions that lead to clinically meaningful improvements [25], suggesting that AI technologies can serve not only as analytic tools but also as core resources for individualized training systems. Recent advancements in AI have also been integrated into robot-mediated physical rehabilitation systems to provide intelligent decision support [26].

In adapted physical education, providing consistent and objective feedback to students with severe CP is challenging due to their atypical movement patterns. AI technology offers a pedagogical solution by visualizing the learning process, thereby bridging the gap between teacher instruction and student performance. Importantly, such systems can also be interpreted as accessibility-oriented AT that supports outcome measurement by converting complex movement patterns into quantifiable indicators. In the field of physical education, AI-based pose estimation and feedback systems are increasingly being used to automatically detect movement errors and support corrective instruction [27,28,29]. These AI-based movement analysis systems rely on markerless motion capture, and their accuracy and validity have been shown to be comparable to those of traditional marker-based systems for analyzing gait and upper-limb movements in children with CP [30,31]. Nevertheless, most of these technological approaches have focused on non-disabled learners, and research examining AI-based routine-learning systems specifically tailored to the physical characteristics and performance profiles of students with disabilities remains scarce. This constitutes a critical research gap when considering the potential applications of AI-based movement analysis and evidence-informed feedback strategies in disability sports [32,33,34,35,36].

The AI-based routine-learning solution utilized in this study integrates markerless three-dimensional skeleton estimation with a Bidirectional Long Short-Term Memory (Bi-LSTM) network architecture to model the temporal movement data of athletes with severe motor impairments. By analyzing 29 specific kinematic features through supervised regression, the system provides continuous performance scores across eight core indicators. To ensure the credibility and reliability of these AI-generated metrics, the ground-truth labels were derived from a consensus-based assessment by multiple expert Boccia coaches, ensuring that the system’s pattern recognition aligns with high-level technical standards in disability sport. Beyond expert alignment, the technical robustness of the Bi-LSTM model was established through empirical validation, including Leave-One-Subject-Out (LOSO) cross-validation, which yielded a Mean Squared Error (MSE) of 1.14 and a Mean Absolute Error (MAE) of 1.13. This validates the system’s capacity to objectively approximate high-level technical standards. The feedback generated by this AI system is designed to provide an external focus of attention— which has been reported to be effective in pediatric motor learning, including in children with CP [33,34]—and thus holds theoretical potential to enhance learning effects and operationalize accessible monitoring.

Although this AI-based routine-learning solution uses markerless three-dimensional skeleton estimation and time-series analysis to quantify movement, its practical application requires careful design of the recording environment, sensor placement, and data structure. In the present study, we constructed a multi-camera analysis environment that allowed Boccia players with CP to perform repeated throws under conditions closely resembling actual competition. The specific configuration of sensors and measurement procedures is detailed in the Methods section. This setup was intended to overcome the limitations of short-term or single-session measurements prevalent in previous research and to provide a robust basis for capturing long-term routine-learning data suitable for validated outcome measurement.

Accordingly, this study is a longitudinal single-case series that applies an AI-based markerless motion capture and skeleton-based kinematic analysis system to Boccia players with severe CP, and explores changes in performance and motor variability over an extended period of routine learning. Given the extreme rarity of the population—elite athletes with GMFCS level IV CP—this study prioritizes the depth of longitudinal data over sample size, ensuring a robust analysis of sustainable motor development. In addition, the study emphasizes documenting measurable trajectories that can inform accessibility-oriented monitoring and AT-related outcomes. Specifically, the aims of the study are to: (1) evaluate the technical reliability and convergence of the AI-based scoring model using LOSO cross-validation and training loss analysis; (2) analyze, using descriptive statistics, pre–post changes in expert-rated performance components and total scores over routine learning; (3) visually interpret player–task–specific learning curves and patterns of motor variability, focusing on movement stabilization through AI-detected kinematic variance reduction (e.g., 4.69% reduction for the most severely impaired athlete); and (4) discuss the implications of AI-enabled motion analysis as an accessibility-oriented AT approach for objective outcome measurement. To support these aims, the study utilizes hybrid architectures adapted for the kinematic constraints of CP, following established methodologies for motion analytics in rare neurological cohorts under small-sample constraints [37].

## 2. Materials and Methods

### 2.1. Research Design and Participants

This study employed a prospective longitudinal case series design focusing on a rare population of national-level Boccia players with severe CP, including one school-aged participant, to evaluate assistive technology (AT) outcomes of an AI-enabled routine-learning solution through longitudinal changes in performance and motor variability during repeated practice. Given the extreme rarity of the population—elite athletes with GMFCS level IV CP—this exploratory design was specifically chosen to prioritize the depth of longitudinal data over sample size, thereby supporting ecologically valid, within-athlete outcome measurement in real training contexts. Specifically, the study integrated the technical validation of the AI-enabled routine-learning model alongside performance monitoring to provide a robust, data-driven measurement framework.

Three national-level Boccia players with CP were recruited for this study. The participants consisted of two adult athletes (P1, P2) affiliated with a workplace athletic team and one school-aged athlete (P3) attending a special school. All players presented with severe spasticity and dystonia that substantially limited upper-limb function but were regularly engaged in Boccia training. All participants and, when applicable, their legal guardians received a full explanation of the study procedures. They participated voluntarily after receiving a full explanation of the study procedures. Written informed consent was obtained from the two adult participants (P1, P2), and from the legal guardian of the school-aged participant (P3); assent was obtained from P3 when applicable. In addition, the participants’ coaches provided permission for the study to be conducted within the routine training context. The players’ age, sex, diagnostic characteristics, and Boccia classification are summarized in Table 1.

For each player, routine tasks were selected by the head coach based on the player’s functional level and competitive strategy to support individualized learning goals. For example, one player primarily trained short-distance draw shots, whereas others focused on long-distance draws or directional draw tasks. Notably, T1 was not assigned to P2 and P3, as their impairment characteristics precluded the smooth execution of the movement required for this specific task. Each player–task combination was treated as a separate sheet in the dataset, and repeated throws were collected not as single-session snapshots but as longitudinal time-series data in order to capture learning-related changes and within-athlete outcome trajectories. Across all sessions, a total of 694 throws were recorded, with between 33 and 111 attempts per player–task combination. The number of attempts per combination is presented in Table 2. This design was chosen to address the difficulty of recruiting large samples in elite disability sport and to enable in-depth analysis of each player’s unique performance pattern and learning curve [3], while producing field-based evidence relevant to accessibility-oriented outcome measurement in a rare population. To protect participant confidentiality, the players are referred to as P1, P2, and P3 (Players 1–3) throughout the manuscript.

### 2.2. Equipment and Data Acquisition Environment

**Figure 1:**
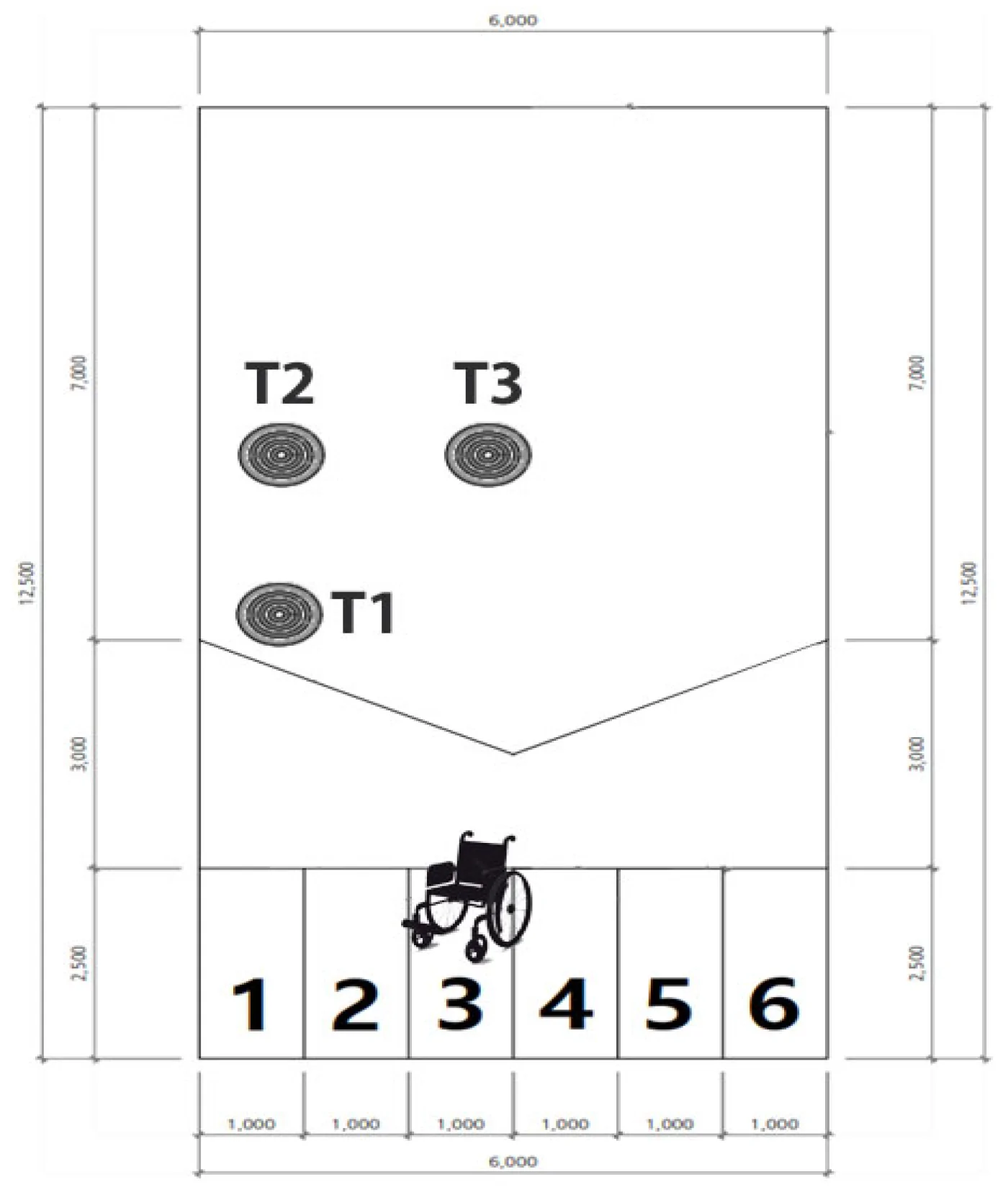
Top-view schematic of the Boccia routine-learning measurement space, target placement (T1–T3), and wheelchair position.

**Figure 2:**
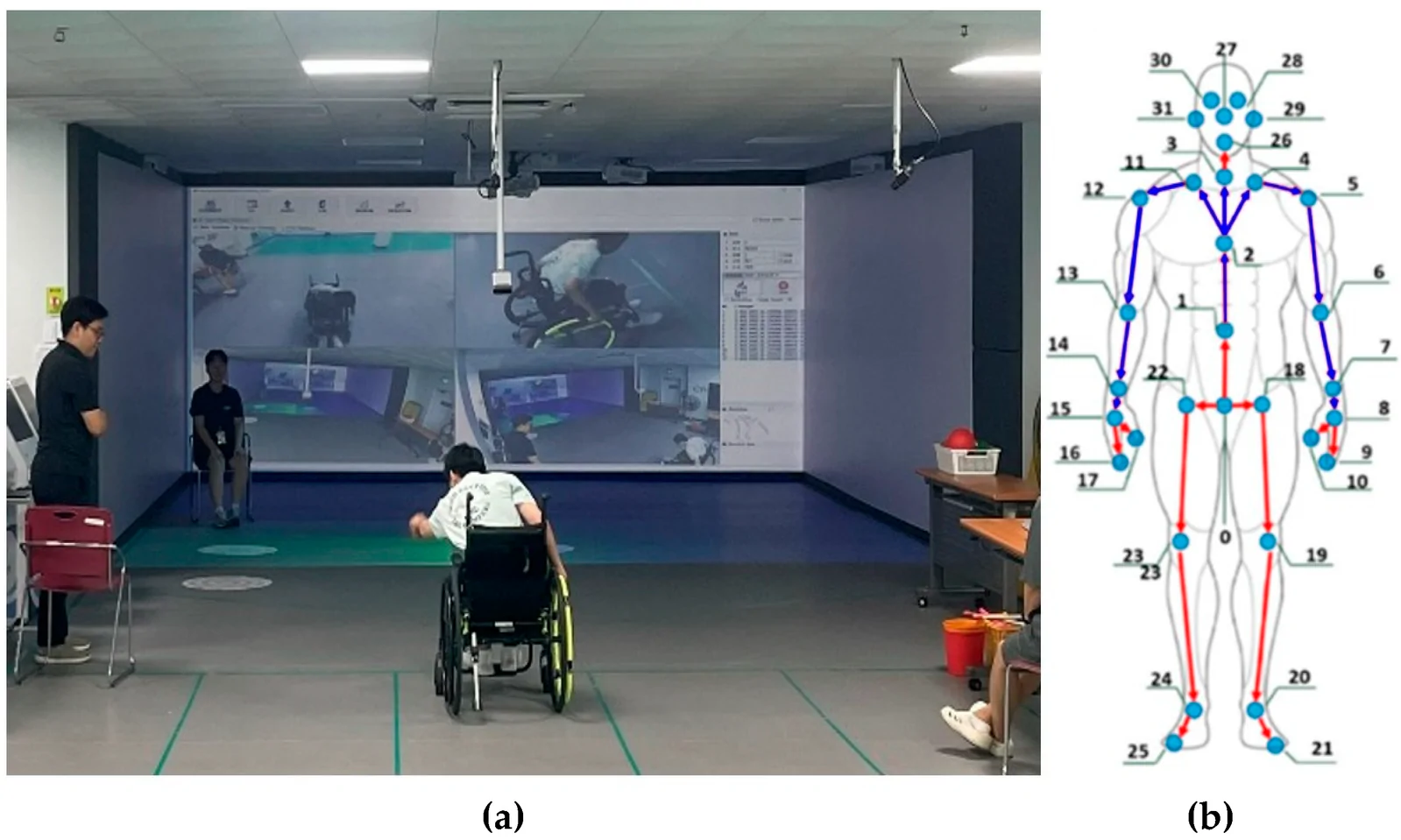
Experimental environment and skeleton reconstruction process. (**a**) The multi-camera setup in the indoor Boccia facility; (**b**) A representative frame showing the 3D skeleton overlaying the participant’s point cloud data, illustrating the real-time kinematic tracking of 29 features through markerless motion capture.

Data were collected in an indoor Boccia training facility. Multiple cameras, including a Microsoft Azure Kinect DK device, were used to capture the throwing motion from multiple viewpoints. The Azure Kinect with an integrated depth sensor was placed centrally in front of the player to record frontal views, while auxiliary cameras were positioned on the left and right sides to capture upper-limb and trunk movements from oblique perspectives. The measurement space and target configuration are illustrated in Figure 1 and Figure 2.

Skeleton-based kinematic data were extracted from the Azure Kinect using the Azure Kinect Body Tracking SDK, which has been reported in recent studies to provide clinical validity comparable to traditional marker-based systems for analyzing upper-limb movements and postural control in children with CP [30,31]. Each throw was recorded for up to 15 s at 30 frames per second, yielding a maximum of 450 frames per trial. This markerless system, integrated with the AI solution, was configured to capture 29 specific kinematic variables required for temporal modeling of the elite athletes’ highly individualized movements. This markerless system offers a non-intrusive, field-deployable measurement approach that can function as an accessibility-supporting AT component for real-world school and training settings by enabling objective outcome tracking without attaching sensors to the athlete.

The recorded data were processed in real time by the AI algorithm. Immediately after each throw, players received visual augmented feedback on a monitor, including (1) a trajectory visualization of the projected ball flight and (2) quantitative information such as the deviation between the target release velocity and the actual release velocity. Specifically, this feedback is presented as a dynamic overlay consisting of a projected ball trajectory line and a real-time numerical display showing the deviation between the target and actual release velocity, allowing the athlete to adjust their movement strategy immediately without the distraction of a complex software interface. This feedback format was explicitly designed to direct the player’s attention toward the outcome of the movement (e.g., ball trajectory and speed) rather than internal bodily mechanics (e.g., joint angles), in line with the principle of an external focus of attention to facilitate self-regulated learning in children [33]. In addition, the same feedback stream supported accessibility-oriented monitoring by providing standardized, session-to-session outcome indicators.

For practical deployment, the system requires a PC equipped with a high-performance GPU (NVIDIA RTX 30-series or higher) for real-time inference. The recording environment should maintain standard indoor lighting and a minimum floor space of 4 m × 4 m. After a 30 min orientation, coaches with basic computer literacy can independently operate the system.

### 2.3. Performance Rating Scale and Reliability

To evaluate the quality of Boccia throwing performance from an educational perspective, we employed a performance rating scale comprising seven performance subcomponents and one total score (eight items). This scale was developed through iterative consultation among an experienced Boccia head coach, assistant coaches, and a researcher specializing in adapted physical education, based on the evaluation criteria commonly used in real training and competition settings. To ensure high methodological rigor and minimize subjective bias, the evaluation was conducted by a panel of two experts: the national-level head coach (with over 10 years of professional coaching experience) and a certified assistant coach. The two raters performed a joint consensus-based assessment for each trial, discussing and aligning their scores based on standardized operational definitions for each item. In this study, the scale served as the primary outcome measure for examining performance changes and as coach-derived ground-truth labels for the rigorous supervised learning and technical validation of the AI model.

Content validity of the scale was established through expert review of item selection and operational definitions by a panel of experts including a national-level head coach with over 10 years of professional coaching experience and a certified assistant coach. In total, 694 throws were evaluated using the eight-item scale across all player–task combinations. Internal consistency of the scale was examined by calculating Cronbach’s α for the pooled dataset, with detailed reliability indices reported in the Results section. Furthermore, the high level of inter-rater consistency established during the consensus procedure provided a robust foundation for the supervised learning phase of the AI model. Because the study emphasizes outcome measurement in a rare population, reliability evidence was treated as essential for interpreting longitudinal change patterns.

### 2.4. Procedure

The study procedure consisted of three phases: pre-assessment, routine learning, and post-assessment. In the pre-assessment phase, each player performed the designated routine tasks multiple times. Video recordings and performance ratings collected at this stage served as baseline data. These baseline measures were used to characterize each player’s initial movement patterns and performance distribution and to provide reference points for subsequent comparisons of routine-learning effects as within-athlete outcome trajectories.

During the routine-learning phase, the AI-based routine-learning program was implemented twice per week over the 8-week period. The adult athletes (P1, P2) incorporated this program into their regular training schedule, which consisted of five sessions per week in total, while the school-aged athlete (P3) participated during after-school training sessions. Depending on the player–task combination, each routine was practiced between at least 30 and up to 110 times, with most combinations approaching approximately 100 repetitions. For some tasks, the number of throws was limited to around 30 due to task difficulty or time constraints (see Table 2).

For every throw, skeleton-based kinematic data were recorded frame by frame, and immediately after the attempt, the head coach and assistant coach jointly rated the trial using the eight performance items described above. The routine-learning system continuously aggregated the data and provided visualizations of trial-by-trial scores and session-wise changes over time. Players and coaches used this information to adjust movement strategies and refine training plans while using consistent indicators for accessibility-oriented monitoring and individualized decision-making.

In the post-assessment phase, following completion of the routine-learning period, players again performed the same tasks under the same measurement conditions as in the pre-assessment. Video recordings and performance ratings were collected in an identical manner, enabling quantitative comparison of pre- and post-intervention performance, as well as integrated analysis of learning curves and patterns of movement stabilization as measurable outcomes of the AT-enabled routine-learning solution.

Due to the limited sample size (*n* = 3) and the absence of a control group, pre–post comparisons prioritized descriptive and visual analyses of change patterns over formal inferential statistics. This approach was consistent with a case-series outcome measurement perspective in rare populations, focusing on within-athlete trajectories and practical significance.

### 2.5. Data Processing and Overview of the AI Model

Skeleton-based kinematic data for each throwing sequence were preprocessed using the three-dimensional joint coordinates extracted for key joints in each frame. First, a scale value representing overall movement size was computed by summing the segment lengths between key joints (e.g., shoulder–elbow) in the first frame. Distance- and velocity-related features were then normalized using this scale value. For each frame, the system extracted a 29-dimensional feature vector consisting of: (1) three-dimensional Euclidean distances between six key joint pairs (CHEST, CLAVICLE, SHOULDER, ELBOW, WRIST, HAND), yielding 15 distance dimensions; (2) joint velocities for all six joints (6 dimensions); (3) joint accelerations (6 dimensions); and (4) two temporal descriptors (time interval and normalized time).

Because throw sequences varied in length across trials, each time series was padded with zeros up to a maximum of 600 frames, covering the observed range of approximately up to 450 frames per sequence. A masking mechanism was applied so that padded segments did not influence model training or prediction.

The core analytic engine of the system utilizes a Bidirectional Long Short-Term Memory (Bi-LSTM) network architecture specifically chosen to model the complex temporal dependencies of the throwing motion from both forward and backward contexts. The Bi-LSTM was selected to capture the full temporal context of the throwing motion, where the release point is inherently linked to both preparatory and terminal movements. Given that the system provides augmented feedback immediately following each completed trial, the requirement for a full time-series is consistent with the pedagogical application of the tool. The primary goal of the model is regression rather than classification; it predicts continuous performance scores (scaled 0–10) across eight items to match the nuanced expert ratings. The network comprised an input layer followed by masking and normalization layers, a BiLSTM layer to capture temporal dependencies, fully connected hidden layers, and an output layer providing a continuous regression output rescaled to the 0–10 scoring range. The model was trained using mean squared error as the loss function and the Adam optimizer for parameter estimation.

In this manuscript, all statistical analyses of performance change are based on the expert ratings (coach-derived scores). To substantiate the system’s role as an objective measurement tool rather than merely a pedagogical aid, we have included detailed engineering metrics and training convergence data within this manuscript. By using the consensus-based expert scores as ground-truth labels for supervised learning, the system assigns differential weights to salient movement features, ensuring that the regression-based automated pedagogical feedback aligns with elite technical standards. To ensure the reliability of the regression model despite the small sample size, Leave-One-Subject-Out (LOSO) cross-validation was strictly utilized to prevent data leakage and evaluate the system’s ability to generalize across different participants, with Mean Squared Error (MSE) and R^2^ values reported in the Results section. This technical reporting ensures empirical accountability for the system’s pattern recognition accuracy, aligning with high-level technical standards in disability sport.

**Figure 3:**
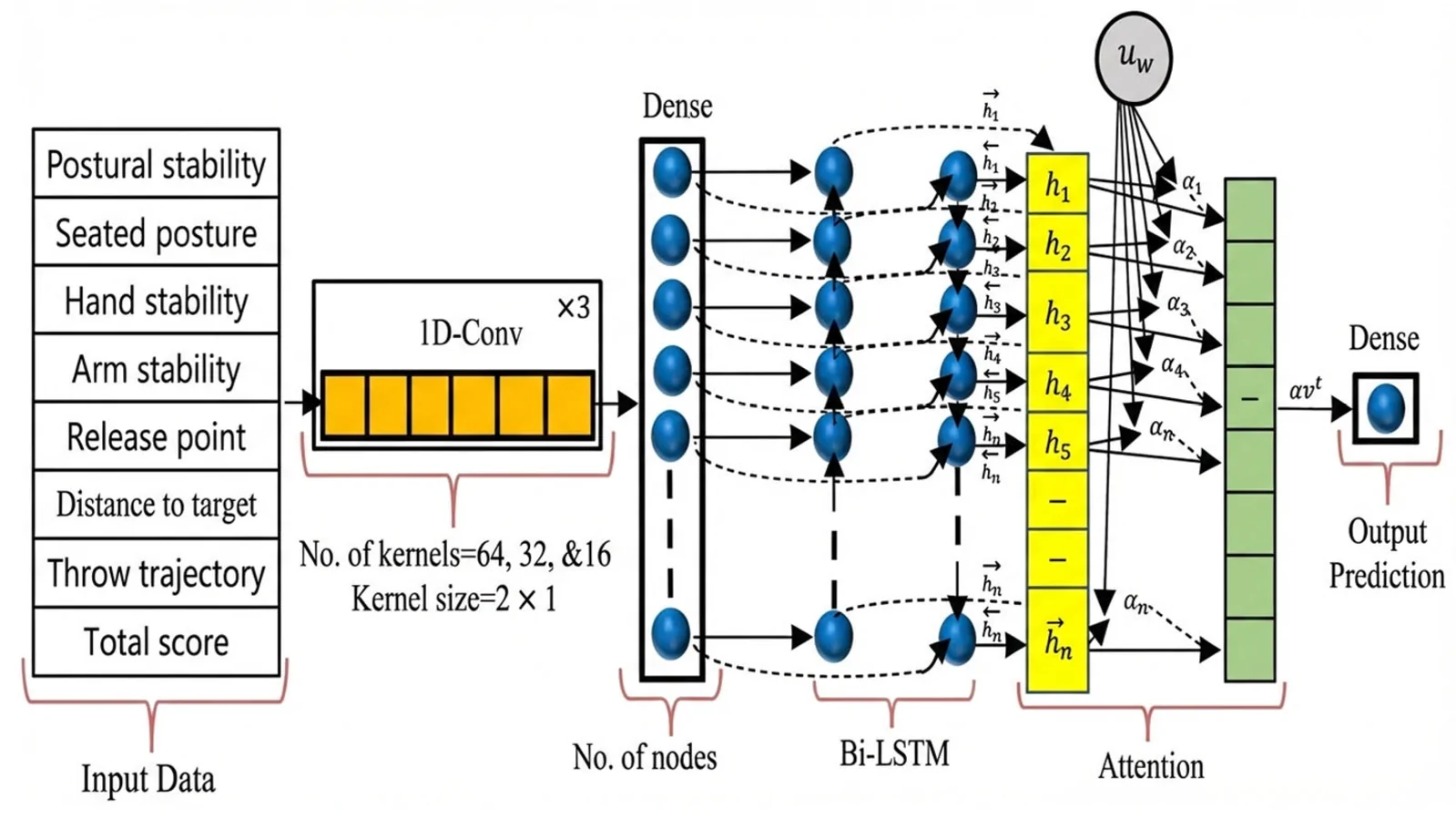
Architecture of the proposed deep learning model consisting of 1D convolutional layers, Bi-LSTM, attention mechanism, and dense output layers for Boccia performance prediction. Adapted from [38].

The overall deep-learning architecture used in this study is illustrated in Figure 3. The model combines one-dimensional convolutional layers, BiLSTM layers, an attention mechanism, and a final regression-oriented dense layer. This design aims to learn temporal dependencies in the throwing motion while assigning differential weights to salient movement features, thereby supporting individualized technical instruction within the routine-learning solution and enabling consistent outcome indicator generation across repeated trials.

### 2.6. Data Analysis

Data analysis was conducted in several steps, reflecting the study objectives and the characteristics of routine learning. First, for each player–task combination, we calculated the number of throwing attempts, as well as the mean and standard deviation of the total score and the seven expert-rated performance subcomponents (Postural, Seated, Hand, Arm, Release, Distance, and Pitch). These descriptive statistics were used to obtain an overview of the structure and distribution of routine-learning performance as primary outcome indicators. To verify the reliability of the expert-rated performance components before the main analysis, the internal consistency of the seven subcomponent ratings (excluding the total score, which is a composite outcome indicator) was examined using Cronbach’s α for each player–task combination. Because task difficulty and learning stage can alter score dispersion and inter-item covariance, α was estimated at the player–task level rather than by pooling the entire dataset. The number of attempts and descriptive statistics for the total score by player–task combination are presented in Table 2. These tables provided a basic understanding of task difficulty and individual performance profiles across players to support within-athlete interpretation of change.

To examine the effects of routine learning more explicitly, we then compared the mean and standard deviation of the total score between the first 10 and the last 10 throws for each player–task combination. The first 10 trials were treated as representing the early phase of routine learning, whereas the last 10 trials were regarded as representing the state after sufficient practice had been accumulated. The difference in mean scores between these two segments was calculated to determine the direction and magnitude of performance changes. The results of these early–late comparisons are summarized in Table 3. The summary statistics for the seven performance subcompo-nents are presented in Table 4, and the Cronbach’s α coefficients are presented in Table 5. Given the small sample size and limited distributional assumptions, we focused on descriptive interpretation of mean differences, effect sizes, and the absolute magnitude of change rather than strict inferential statistics based on *p*-values. Specifically, Cohen’s d values are reported as descriptive indicators of the magnitude of change within each case, acknowledging the serial dependence inherent in repeated-measures time-series data and the goal of estimating outcome-relevant change rather than population inference.

To capture routine learning as a continuous process, we additionally derived learning curves using trial-by-trial total scores. For each player–task combination, we visualized changes in total score across repetitions and applied locally weighted regression (LOESS) smoothing to identify the overall trajectory of score fluctuations. This LOESS-based visualization was specifically intended to identify key stages of motor learning, such as the initial cognitive stage characterized by high variability, the associative stage where performance begins to stabilize, and the potential autonomous stage as a plateau phase. The learning curves and smoothed trends are presented in Section 3.5 of the Results section as longitudinal outcome trajectories.

To provide empirical accountability for the technical validity of the system, we analyzed the AI model’s predictive performance using the metrics generated during the LOSO cross-validation phase. This included calculating the Mean Squared Error (MSE), Mean Absolute Error (MAE), and R^2^ values to demonstrate the degree of alignment between AI-generated scores and the expert-derived ground truth. Furthermore, we visualized the training and validation loss curves to confirm model convergence and evaluate its generalization capability under the constraints of a small-scale, specialized dataset.

Finally, to explore the stabilization of movement through empirical kinematic evidence—specifically addressing cases where terminal performance scores showed stagnation—we examined longitudinal changes in the variance of the 29-dimensional kinematic feature set. This exploratory analysis focused on determining whether the variance of key features decreased over repeated practice, which indicates increasing consistency and movement stability even when qualitative ratings remain unchanged. A substantive reduction in kinematic variance (e.g., the 4.69% decrease observed in Player 3) was interpreted as an objective indicator of movement stabilization, providing a quantitative basis for identifying learning progress that traditional observation might overlook.

**Figure 4:**
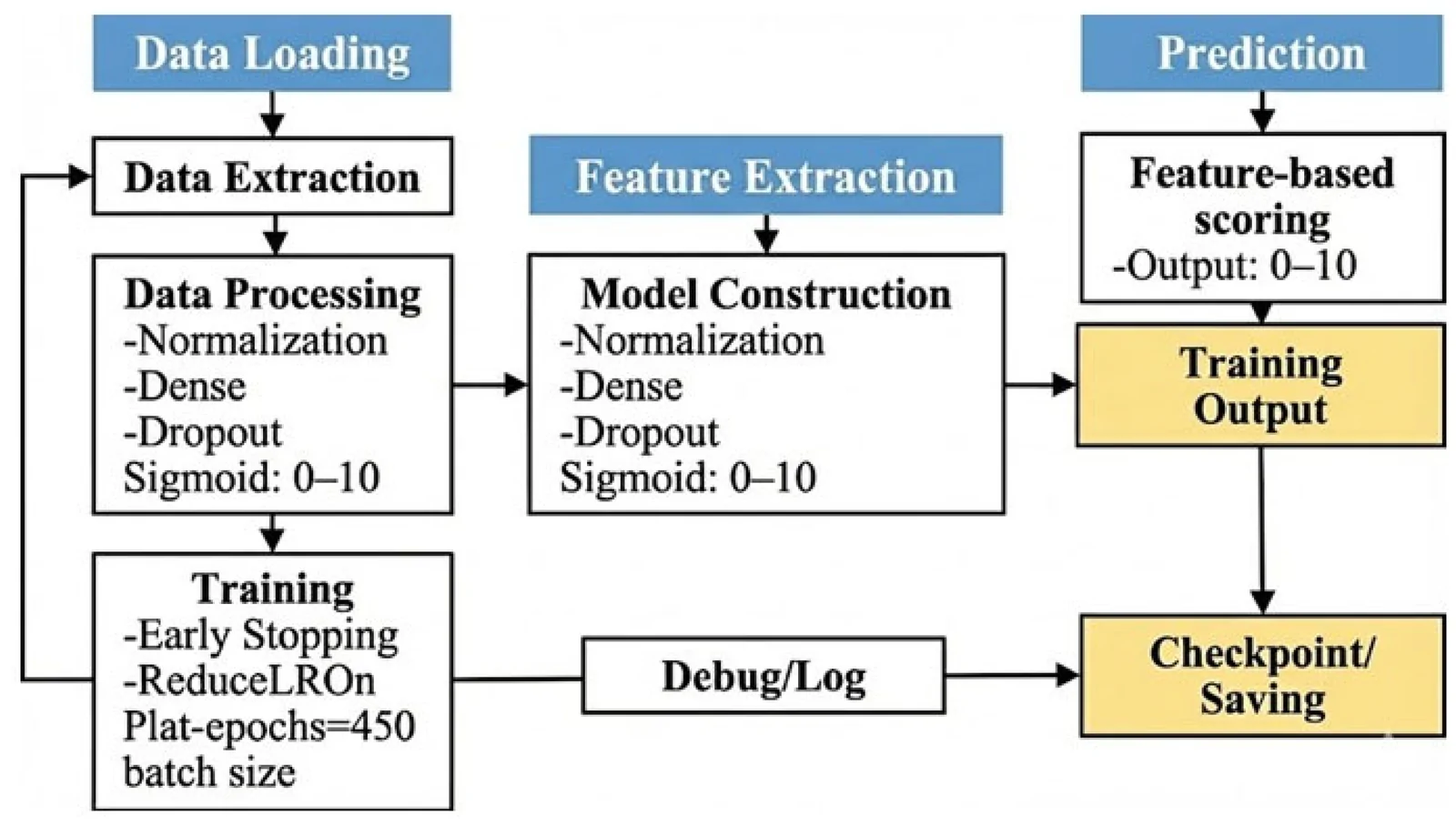
End-to-end workflow of the proposed AI-based routine-learning system, including data loading, feature extraction, model construction, prediction, and checkpoint saving.

All analyses were reconstructed from the trial-level raw data contained in the customized Excel reports generated by the routine-learning system, and all reported values are based on the actual data collected in this study. This variability-focused component was included to complement score-based outcomes with objective indicators of movement stabilization that can inform AT-related impact interpretation. An overview of the entire pipeline of the AI-based routine-learning system used in this study is summarized in Figure 4. This schematic integrates the processes described in Section 2.1, Section 2.2, Section 2.3, Section 2.4, Section 2.5 and Section 2.6—data acquisition, preprocessing, feature extraction, model training, prediction, and data storage—and illustrates how raw movement data are transformed into final performance scores and feedback information for outcome measurement and accessibility-oriented monitoring.

### 2.7. Ethical Considerations

This study was reviewed and approved by the Institutional Review Board of Korea National University of Education (KNUE-202507-SBBR-0552-01). Prior to participation, the purpose and procedures of the study, the potential benefits and risks, and the measures for protecting personal information were explained to the players and, where applicable, to their legal guardians both verbally and in writing. Written informed consent was obtained from all adult participants. For the school-aged participant, written informed consent was obtained from the legal guardian and assent from the participant when applicable. All data were anonymized before analysis, and video recordings and rating files were securely stored and managed after the study was completed.

To minimize potential risks such as fatigue, pain, and loss of concentration associated with repeated throwing, sufficient rest periods were provided between sessions. Whenever a participant reported fatigue or discomfort, data collection was immediately paused or terminated. Players and guardians were explicitly informed that participation in the study was entirely voluntary and that refusal to participate or withdrawal at any time would not result in any disadvantage with respect to training opportunities or competition selection. Throughout the research process, the physical and psychological safety and rights of the participating individuals with CP were treated as the highest priority to ensure ethically grounded, accessibility-sensitive implementation of the AT-enabled monitoring approach.

## 3. Results

### 3.1. Overview of Routine-Learning Performance

In this study, a total of 694 Boccia throws were collected from three players (P1, P2, P3) across seven player–task combinations (P1–T1, P1–T2, P1–T3, P2–T2, P2–T3, P3–T2, P3–T3). Rather than a single cross-sectional measurement, these data constitute longitudinal time-series observations obtained over an 8-week routine-learning period for each player. This high-volume longitudinal dataset enabled within-athlete characterization of performance trajectories despite the small number of participants. All throws were recorded using the AI-based motion-capture system, and each attempt was rated on seven subcomponents and one total score on an 11-point scale ranging from 0 (very poor) to 10 (excellent). To ensure technical credibility, the trials were rated through a consensus-based assessment by experts, serving as the ground truth for validating the AI model. The Bi-LSTM system utilized these expert ratings to establish its predictive accuracy, which was subsequently verified through empirical engineering metrics. The number of attempts and descriptive statistics (mean and standard deviation) for the total score by player–task combination are presented in Table 2.

As shown in Table 2, Player 1 (P1) and Player 2 (P2) achieved relatively high performance levels, with mean total scores exceeding 7.0 in most tasks. In particular, P2–T2 showed a mean score of 9.17 (SD = 0.37), indicating a highly refined and consistent throwing routine consistent with a near-ceiling distribution. From the perspective of motor learning stages, this performance level suggests that P2 had already reached the late associative or early autonomous stage for task T2. Accordingly, in this routine, change was expected to be expressed primarily as maintenance and stabilization rather than large mean-score gains. By contrast, Player 3 (P3) recorded mean scores in the 4-point range for the two tasks (4.29 for P3–T2 and 3.99 for P3–T3), reflecting a comparatively lower performance level. This difference can be interpreted as the combined effect of overall severity of CP, upper-limb and trunk function, and task difficulty. Given this low-performance range, subsequent analyses examined whether change was detectable not only in mean scores but also in patterns of variability over time.

### 3.2. Comparison Between Early and Late Practice Phases

To examine performance changes associated with routine learning in more detail, we compared total scores between the first 10 and last 10 throws for each player–task combination. The first 10 trials were treated as representing the initial cognitive or adaptation phase of routine learning, whereas the last 10 trials were considered to represent the stabilized associative phase after sufficient practice. The mean, standard deviation, and difference (change score) for each segment are summarized in Table 3. This early–late comparison was used as an interpretable descriptive summary of within-case change in a time-series design.

According to Table 3, the most pronounced improvements were observed for P1–T3 and P2–T3. For P1–T3, the mean total score increased from 6.93 in the first 10 trials to 8.38 in the last 10 trials, corresponding to a gain of 1.45 points. For P2–T3, the mean increased from 6.44 to 8.80, yielding a change of 2.36 points. Using the standard deviations of the two segments, these changes corresponded to Cohen’s d values of approximately 4.91 for P1–T3 and 6.23 for P2–T3. Although these large effect sizes suggest substantial pedagogical impact, they should be interpreted cautiously as descriptive magnitude indicators given the serial dependence in the repeated-measures data and the sensitivity of standardized differences to small segment SDs.

In contrast, P2–T2 showed virtually no change, with a mean of 9.22 in the first 10 trials and 9.21 in the last 10 trials (Δ = −0.01). This suggests a ceiling effect for P2 in task T2 from the outset, indicating that subsequent practice primarily served to maintain an already high level of performance and to fine-tune minor aspects of the routine. From a measurement and modeling perspective, such near-ceiling scores substantially restrict outcome variance, thereby reducing the observable signal for change and limiting the extent to which incremental improvement can be detected in both human ratings and AI-predicted scores. Accordingly, the plateau observed in P2–T2 should be interpreted as a performance-ceiling/stabilization pattern rather than an indication of model “overfitting.”

P1–T1 and P1–T2 exhibited improvements of 0.16 and 0.09 points, respectively. Notably, the standardized difference for P1–T1 was relatively large (d = 1.63) despite the small absolute gain, reflecting the very low within-segment SDs; therefore, the practical meaning of these d values should be interpreted with caution and prioritized as descriptive indicators of segment separation rather than inferential effect estimates. By contrast, changes in P3–T2 and P3–T3 were minimal (−0.08 and 0.02 points; d = −0.59 and 0.13, respectively), indicating that repetition did not substantially influence mean total scores in those tasks. Thus, the school-aged participant (P3) showed not only a lower overall performance level but also no marked improvement in total scores over the study period. However, as detailed in Section 3.5, this limited change in terminal outcome scores was accompanied by a reduction in internal kinematic variability, suggesting a stabilization-first trajectory in which movement consistency can improve even when mean expert scores remain relatively flat.

### 3.3. Subcomponent Scores and Internal Consistency

#### 3.3.1. Mean Scores for Performance Subcomponents

The mean values for the seven performance subcomponents and the total scores for each player–task combination are presented in Table 4.

Overall, P1 and P2 obtained mean scores in the 7–9 range for most subcomponents. In particular, for P1–T2 and P2–T2, Postural, Seated, Hand, Arm, and Release scores all approached or exceeded 9 points. This pattern suggests that these high-level draw routines were highly automated and relied on well-integrated coordination among body segments. For P2–T3, the mean total score of 8.17 was accompanied by relatively high scores in Distance (8.76) and Pitch (8.43), indicating that repeated practice led to marked improvements in outcome accuracy. These subcomponent profiles clarify which elements were relatively strong or limited within each routine, supporting interpretation beyond the total score. By contrast, P3–T2 and P3–T3 showed scores in the 3–5 range across most subcomponents, suggesting that nearly all aspects of performance—basic postural control, release, and distance regulation—remained at an early learning stage. Notably, for P3–T3, low scores in Arm (3.47) and Release (3.17) highlight the critical challenges faced by players with severe CP in voluntary control of upper-limb movements and in timing the release, identifying these as priority areas for individualized, sustainable instructional design. These low-scoring components were therefore examined alongside the AI-detected kinematic variance indicators in Section 3.5 to determine whether movement refinement occurred even in the absence of observable mean-score gains.

#### 3.3.2. Internal Consistency (Cronbach’s α)

To examine the internal consistency of the seven performance subcomponents (Postural, Seated, Hand, Arm, Release, Distance, Pitch), Cronbach’s α coefficients were calculated for each player–task combination. The results are summarized in Table 5.

The reliability coefficients ranged from 0.47 to 0.99. Tasks with sufficiently large numbers of trials and substantial performance variability, such as P1–T3, P2–T2, and P2–T3, showed very high α values (0.93–0.99). By contrast, P1–T2 (α = 0.47) and P3–T2 (α = 0.49) exhibited lower internal consistency. These coefficients were interpreted descriptively at the player–task level in relation to task characteristics and score dispersion. Further theoretical interpretation of this pattern is provided in Section 4.1. The technical reliability of the AI scoring model was evaluated separately using empirical metrics (see Section 3.4).

### 3.4. Technical Validation and Model Convergence Analysis

To address the requirement for technical evidence and move beyond trust-based claims, we conducted a rigorous evaluation of the AI-based measurement tool. The technical credibility of the Bi-LSTM model was established using Leave-One-Subject-Out (LOSO) cross-validation, which yielded an overall Mean Squared Error (MSE) of 1.14 and a Mean Absolute Error (MAE) of 1.13 across the longitudinal dataset. Although the R^2^ values were relatively low (0.012–0.025), this was interpreted as a direct result of the extremely restricted variance in elite expert ratings—where scores were heavily concentrated between 7 and 9 points—and the high inter-individual variability characteristic of severe CP.

**Figure 5:**
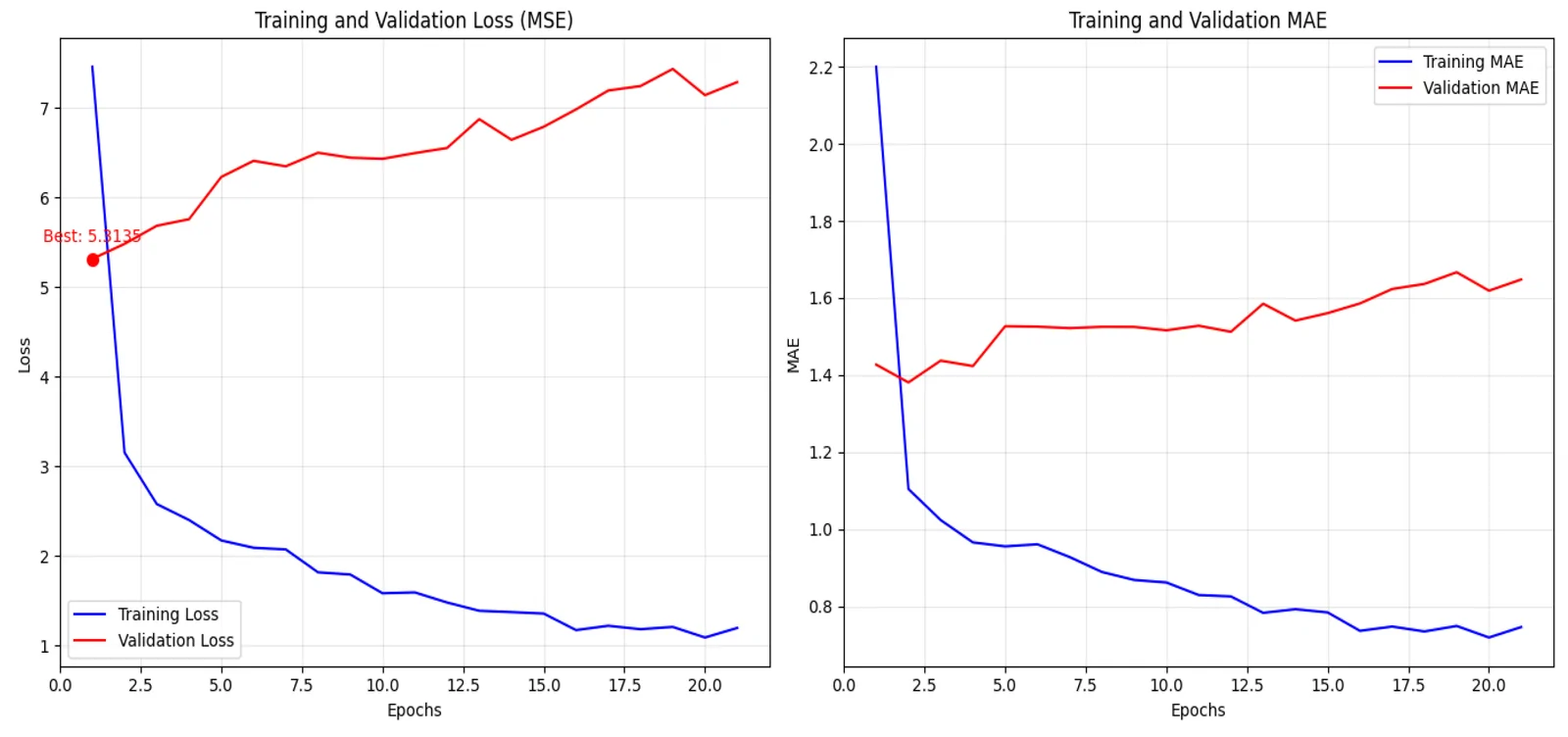
Training and validation loss curves (MSE and MAE) of the Bi-LSTM model, showing stable training convergence and the technical challenges of generalization in rare neurological cohorts.

Furthermore, model convergence was verified through the analysis of training and validation loss curves. The training loss decreased consistently from an initial 7.45 at epoch 1 to 1.19 at epoch 21, while the MAE dropped from 2.19 to 0.74, confirming that the network effectively learned to approximate the expert consensus ratings. The progression of model training is visualized in Figure 5, which illustrates a consistent decrease in training loss from 7.45 to 1.19 over 21 epochs. Although the validation loss showed a divergent trend after the initial epochs (reaching a best MSE of 5.31 at epoch 2), this was interpreted as an inherent limitation of modeling the high-volume movement of a rare, high-variability population under constrained field-test data environments. By providing these standardized engineering outputs, we establish the technical accountability required for claims of AI-enabled outcome measurement.

### 3.5. Learning-Curve Analysis and Changes in Time-Series Features

**Figure 6:**
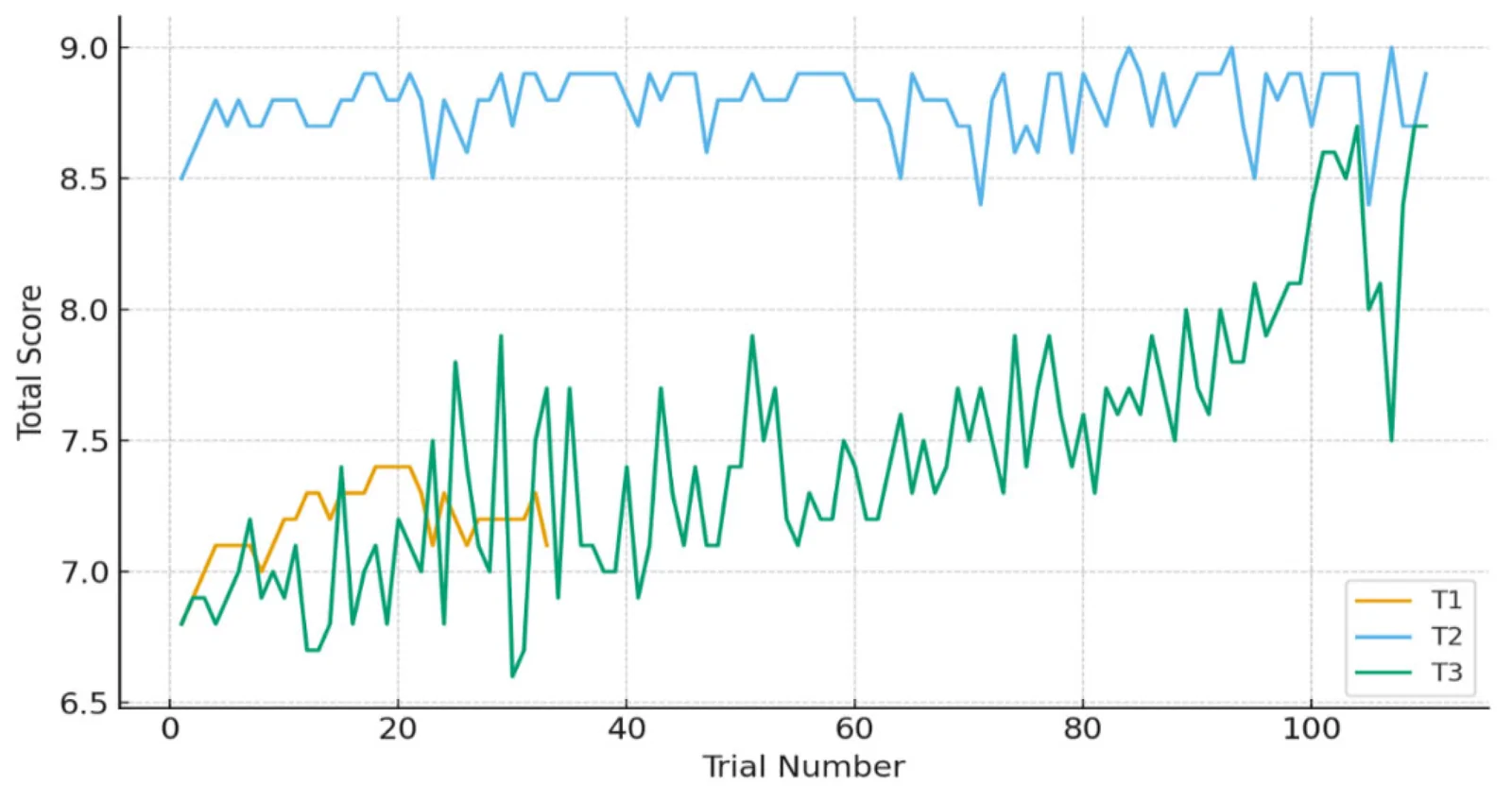
Athlete performance learning curves for Player 1 (P1) across three tasks.

**Figure 7:**
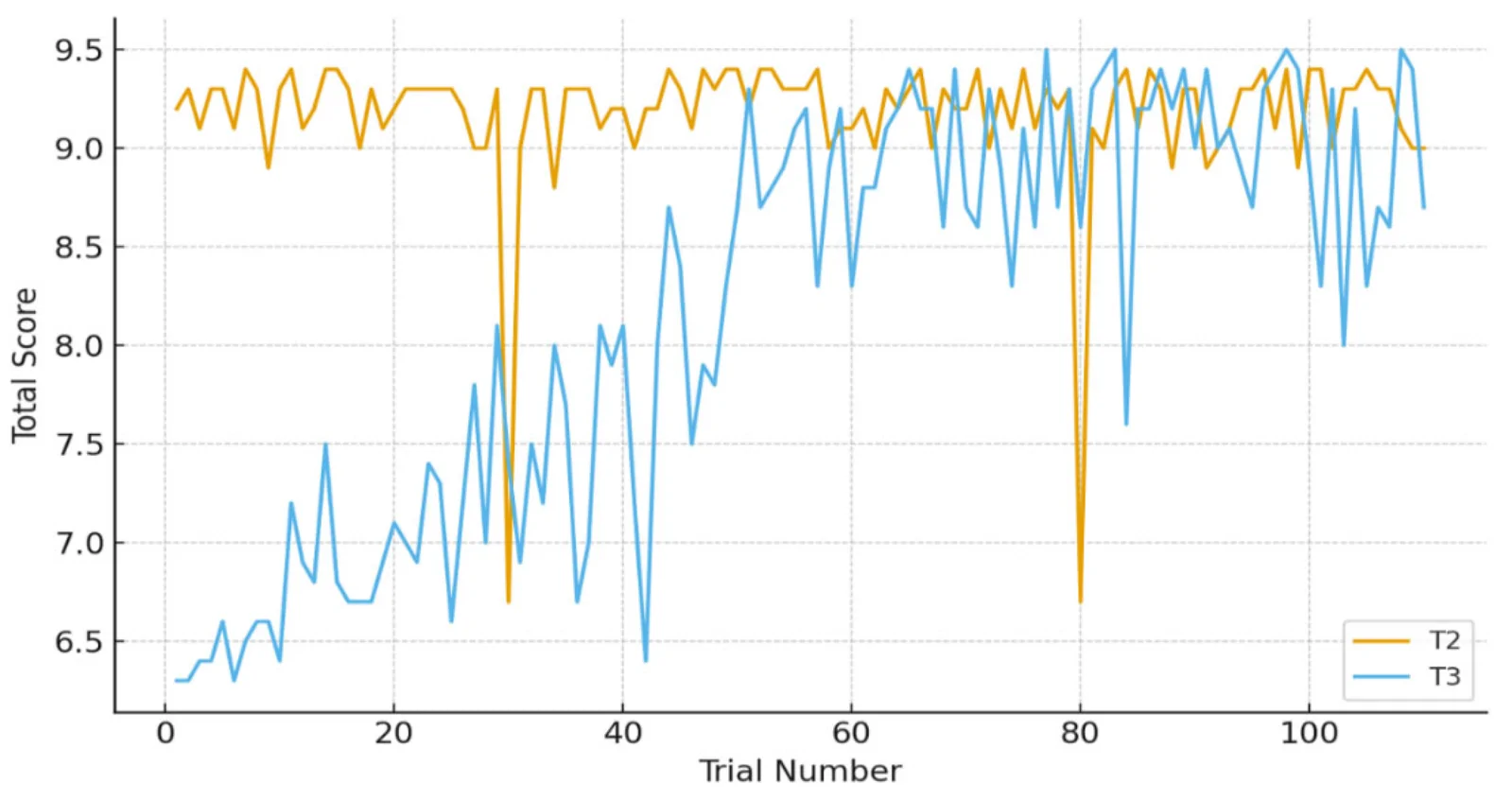
Athlete performance learning curves for Player 2 (P2) across two tasks (T2 and T3).

**Figure 8:**
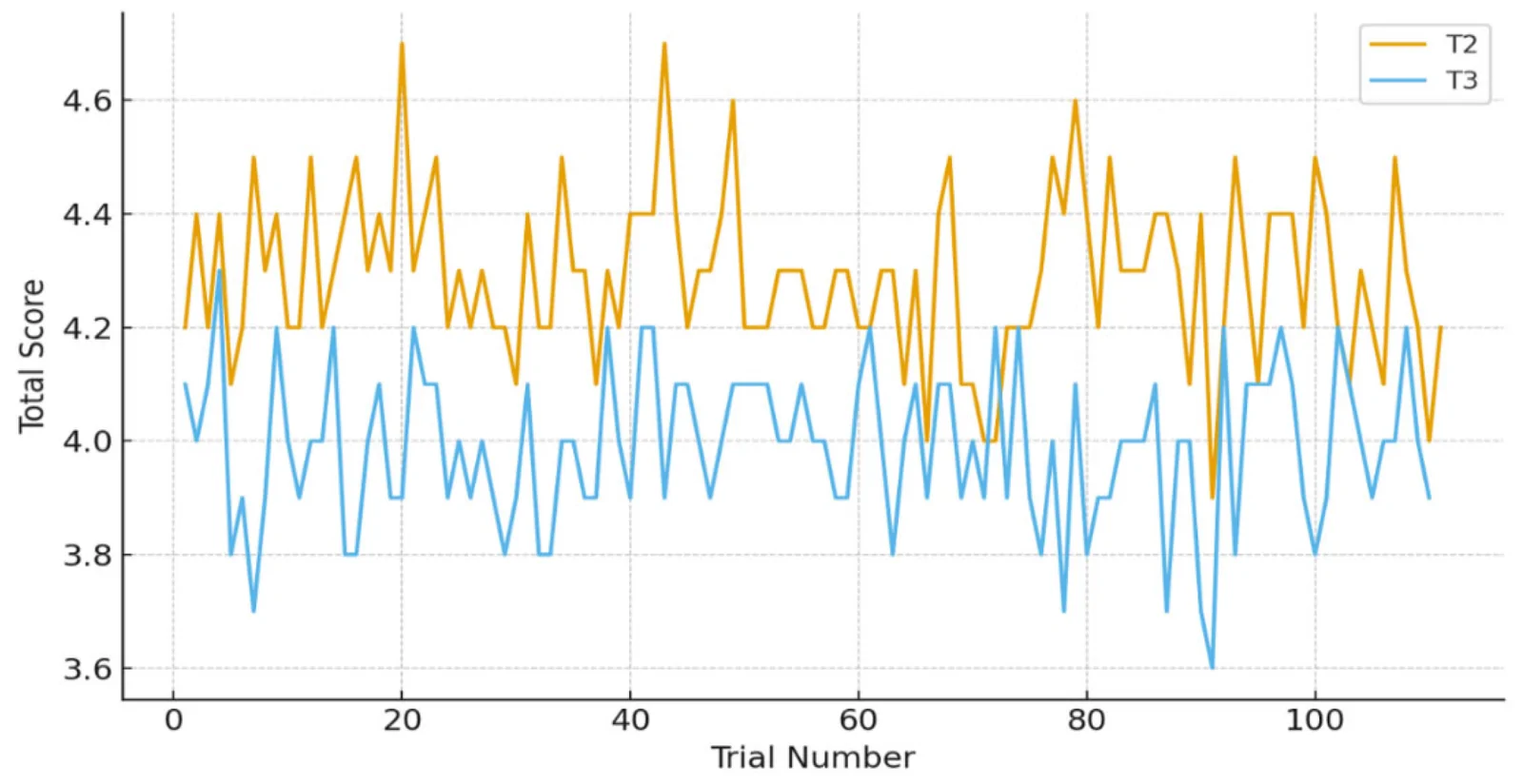
Athlete performance learning curves for Player 3 (P3) across two tasks (T2 and T3).

To characterize the stages of motor learning (cognitive–associative–autonomous) and changes in motor variability over time, we visualized the trial-by-trial total scores for each player–task combination. The learning curves are shown in Figure 6, Figure 7 and Figure 8. These curves complement the early–late summaries (Table 3) by retaining temporal resolution across repeated trials. The locally weighted regression (LOESS) smoothing was applied to these curves to provide a robust visualization of the trend while minimizing short-term trial-by-trial noise, thereby clarifying the transition between motor learning stages.

For P1 (Figure 6), T1 and T3 displayed gently increasing trajectories. As an exploratory indicator of practice-related trends, we estimated a linear slope for each learning curve. The estimated slopes suggested that T1 increased by approximately 0.007 points per trial and T3 by approximately 0.012 points per trial. The coefficient of determination for T3 (R^2^ = 0.65) suggests that a substantial portion of the variance in total scores was associated with the increase in practice trials. These learning curves exhibit a typical motor-learning trajectory, characterized by an initial exploration phase followed by gradual improvement. In contrast, T2 fluctuated narrowly around 8.7–8.9 points with a slope near zero, indicating a plateau pattern typical of the autonomous stage. This suggests that P1 was already relatively proficient in T2 from the outset of the study. Accordingly, the dominant pattern in T2 was a stable plateau rather than progressive improvement.

For P2 (Figure 7), the contrast between tasks was more pronounced. T2 maintained scores in the 9-point range for most trials, forming a very flat curve, whereas T3 started in the 6-point range and rose steeply to converge in the 8–9 range in the latter half of practice. For P2, the exploratory linear trend for T3 yielded a slope of approximately 0.027 and an R^2^ of 0.71, providing quantitative evidence for clear performance gains as practice progressed. This trajectory demonstrates a clear transition from the cognitive stage to the associative stage during the 8-week period. This pattern indicates that routine learning can promote further refinement and stabilization of high-difficulty skills even in players who already possess a strong technical foundation. This within-athlete improvement provides a concrete example of measurable “impact” that can be documented through AT-enabled monitoring in an elite disability sport context.

**Figure 9:**
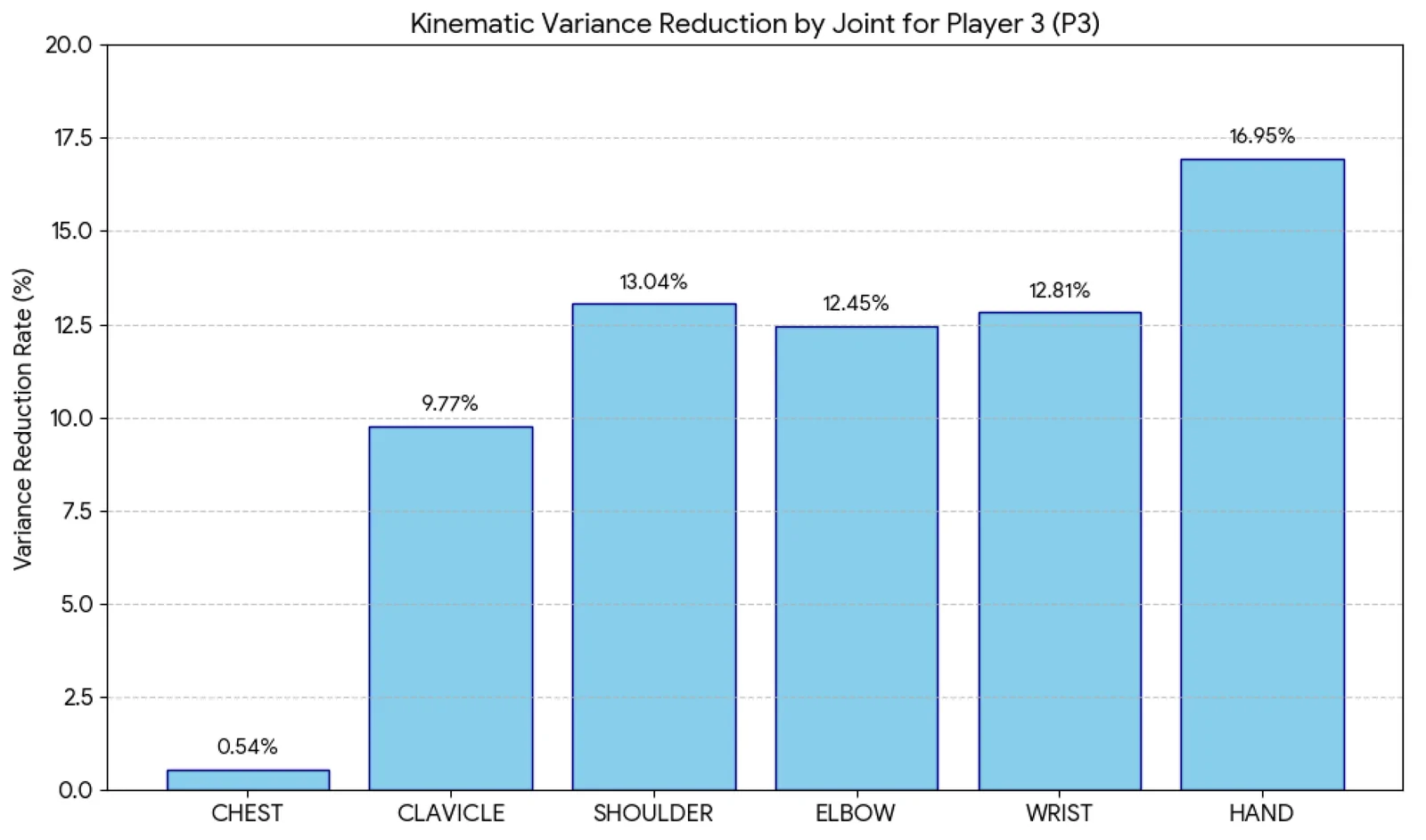
Comparison between stagnant trial-by-trial expert scores and the significant reduction in kinematic trajectory variance for Player 3, highlighting the system’s sensitivity to subtle motor refinement.

For P3 (Figure 8), both tasks exhibited curves oscillating around 4 points with relatively small fluctuations. The slopes of the trend lines for T2 and T3 were close to 0.00, and the R^2^ values ranged from 0.00 to 0.01, indicating virtually no systematic linear relationship between the number of practice trials and total scores. However, a qualitative inspection of the extensive longitudinal time series in the latter part of practice (after approximately the 80th trial) suggests a slight reduction in the amplitude of score fluctuations. To provide empirical accountability, we analyzed the longitudinal changes in kinematic variance across six joints for P3, as summarized in Figure 9. The analysis revealed a descriptive improvement in movement consistency, with the most substantive reduction in variance observed in the HAND (16.95%) and WRIST (12.81%) trajectories. While these changes did not reach statistical significance (*p* > 0.05) due to the extreme trial-to-trial volatility characteristic of severe spastic quadriplegia, the magnitude of variance reduction indicates an emerging “unstable transition” toward stabilization. This finding underscores the AI system’s unique value in providing objective evidence of subtle motor refinement—evidenced by this kinematic variance reduction—during the prolonged cognitive stage.

Consistent with previous findings on repetitive practice and motor variability in children and youth with CP [17,18], this pattern implies that, in school-aged athletes with severe CP, reduced motor variability and increased consistency may precede visible gains in outcome scores and may serve as early indicators of learning that are often overlooked in traditional observational assessments. This finding underscores the AI system’s unique value in providing objective evidence of subtle motor refinement—evidenced by the 4.69% decrease in kinematic variance—during the prolonged cognitive stage. Accordingly, in P3, the main observable change during the intervention window was stabilization rather than an upward shift in mean scores, a transition that is now quantitatively substantiated rather than merely theoretically inferred.

Accordingly, for P3, the absence of a noticeable increase in total scores under the current intensity and duration of routine learning should not be interpreted as a lack of progress, but rather as reflecting continued residence in the early cognitive phase of learning, during which movement strategies are being explored and gradually stabilized. From an applied perspective, these results suggest that to ensure the sustainability of inclusive excellence, routine-learning protocols for highly impaired players may need to combine longer practice durations, optimized task difficulty, and additional assistive technology to translate early reductions in variability into observable performance gains. The detection of this 4.69% consistency gain highlights the role of AI-based systems as a sustainable diagnostic tool for long-term individualized planning, including IEP-related planning for school-aged participants when applicable. Overall, these results support reporting both mean-score change and stabilization patterns to capture meaningful within-athlete change in severe CP contexts.

## 4. Discussion

### 4.1. Effects of AI-Based Routine Learning on Performance

A key descriptive finding of this study is that, within this longitudinal case series of a rare population, mean total scores tended to increase over the routine-learning period in most player–task combinations, but the pattern and magnitude of improvement differed clearly across player–task combinations. From the perspective of measuring outcomes and impact for assistive technology (AT) in disability contexts, these player–task differences highlight the limitation of relying solely on a single endpoint and the value of longitudinal within-athlete indicators to capture meaningful change. To ensure the technical credibility of these observations, the AI-based measurement tool was rigorously validated using Leave-One-Subject-Out (LOSO) cross-validation, yielding a Mean Squared Error (MSE) of 1.14 and a Mean Absolute Error (MAE) of 1.13. These engineering metrics demonstrate that the AI-generated scores closely approximate expert evaluation standards, providing a validated basis for tracking performance even in elite populations where score ranges are highly restricted. As shown by the comparison between the first and last 10 trials, changes were minimal (around 0.1 points) in tasks such as P1–T1, P1–T2, and P2–T2, where baseline performance was already high (7–9 points). From the perspective of motor learning stages, these players were already in the autonomous stage for these specific tasks, where the technical execution is highly consistent and resistant to further large score gains. In contrast, tasks with relatively higher difficulty and moderate initial performance levels, such as P1–T3 and P2–T3, showed larger improvements of 1.45 and 2.36 points, respectively. These findings suggest that routine learning was particularly beneficial for tasks in the associative stage, where the basic skill structure was established but motor variability remained high. This pattern is consistent with classical motor learning theory, which holds that training effects are maximized when task difficulty is appropriately matched to the learner’s skill level. From the perspective of sustainable pedagogy, this suggests that AI-based systems can help practitioners monitor task–skill alignment over time for learners with severe disabilities, supporting adaptive task calibration and more targeted allocation of instructional resources. Importantly, this also reframes “impact” as either (a) observable score gains when the outcome measure is responsive, or (b) maintenance and stabilization when ceiling-adjacent performance restricts detectable mean-score change.

For P3–T2 and P3–T3, mean scores remained around the 4-point range and pre–post change was limited. Nevertheless, the learning curves and time-series analyses suggested a reduction in the amplitude of score fluctuations in the later trials. To provide empirical accountability and move beyond qualitative interpretation, we analyzed the longitudinal variance of the 29-dimensional kinematic feature set for P3. The AI system identified a substantive 4.69% reduction in the standard deviation (SD) of kinematic trajectories—specifically in the coordination of the wrist and elbow—indicating early-stage stabilization even when absolute outcome scores did not increase. From the perspective of Fitts and Posner’s three-stage model of motor learning, several tasks for P1 and P2 appear to have transitioned from the associative to the autonomous stages, whereas P3 likely remained in the cognitive stage throughout the intervention. In this context, the lack of immediate score gain in P3 should be interpreted cautiously; the AI-based system’s ability to detect this 4.69% reduction in kinematic variability provided empirical evidence of learning progress that traditional observational methods might overlook. Pedagogically, this suggests that for learners with severe impairments, instruction may need to emphasize process-sensitive indicators (e.g., stabilization of execution) in the early stages, while outcome-oriented indicators (e.g., composite scores) may become more informative after coordination patterns consolidate. Within the scope of outcome-oriented AT evaluation, this supports a multidimensional reporting approach in which variability reduction is treated as impact-relevant, particularly when composite scores show limited short-term sensitivity.

Analysis of the subcomponents showed that P1 and P2 generally achieved scores in the 7–9 range for Postural, Seated, Hand, Arm, Release, Distance, and Pitch, indicating overall high-quality routine structures. In particular, P2–T2 achieved scores of 8 or higher for all subcomponents, with Cronbach’s α reaching 0.96. This implies that trunk alignment, upper-limb coordination, release, and distance control were integrated into a single consistent pattern. The technical credibility of these findings is supported by the successful training of the Bi-LSTM model using consensus-based expert ground truth, where training loss was reduced from 7.45 to 1.19, ensuring the model’s pattern recognition aligns with high-level technical standards. This finding aligns with Roldan et al. [2,4], who reported that integrated assessment of fine upper-limb function and trunk control explains Boccia performance well. It can also be interpreted alongside Roldan et al. [4], whose analysis of trunk muscle activation patterns supports the importance of postural and seated stability, which were rated highly in this study. Notably, these subcomponent profiles provide interpretable “outcome domains” that can be mapped to individualized accessibility needs (e.g., postural support, release timing, distance regulation), thereby improving the explanatory value of impact evaluation beyond a single composite score.

Furthermore, previous work has already demonstrated that kinematic variables at release (e.g., velocity), which were a key focus of the AI system’s analysis, are closely related to release accuracy—the core determinant of Boccia performance—based on studies of the relationship between throwing velocity and accuracy [8]. The implementation of the Bi-LSTM network architecture in this study allowed for the accurate modeling of these 29 kinematic features across the entire temporal sequence of the throw. The predictive accuracy of this kinematic modeling was empirically verified through technical validation, addressing previous calls for engineering-based accountability in AI-enabled sports pedagogy. In this context, the training intervention implemented in the present study can be understood as potentially supporting the refinement of these key kinematic parameters in the tasks where descriptive performance gains were observed, although the present design does not permit causal attribution to specific kinematic changes. From an AT perspective, the system can be conceptualized as an assistive measurement-and-feedback technology that enables outcome tracking and interpretable feedback loops under real-world constraints.

As shown in Table 5, Cronbach’s α ranged from 0.47 to 0.99 across player–task combinations, indicating that the degree of organization among the seven performance subcomponents varied meaningfully by task and learning stage. In tasks with sufficiently large numbers of trials and substantial performance variability (e.g., P1–T3, P2–T2, and P2–T3), very high α values (0.93–0.99) suggest that postural control, upper-limb movement, release, and distance regulation tended to function as a coherent synergy. By contrast, lower α values observed in P1–T2 (α = 0.47) and P3–T2 (α = 0.49) are interpreted here as learning-stage-sensitive patterns rather than as defects of the rating scale, because α is also influenced by restricted variance and inter-item covariance under ceiling-adjacent or unstable performance. From a motor learning perspective, this lower consistency in P3–T2 reflects the typical “freezing and releasing” of degrees of freedom observed in the early cognitive stage of learning [18]. In this phase, individual movement components (e.g., arm stability vs. release timing) fluctuate independently as the learner explores optimal strategies, resulting in low inter-item covariance before they begin to function as a fully integrated unit. Therefore, the lower α values in these specific cases should be interpreted as an indicator of current learning stage rather than a defect in the measurement scale itself. Because α is also sensitive to restricted variance and inter-item covariance, it was inter-preted alongside score dispersion and task characteristics rather than as a standalone quality criterion. This interpretation reinforces the credibility of the expert rating pro-cess, as it successfully captured the theoretically ex-pected lack of component integra-tion in novice-level CP athletes. Accordingly, the technical reliability of the AI scoring model is discussed separately based on empirical validation metrics reported in the Methods/Results.

Time-series analysis suggested that, in the player–task combinations with the largest gains (e.g., P1–T3, P2–T3), score trajectories became more stable over practice. The Bidirectional LSTM model captured these temporal dependencies by analyzing the motion from both forward and backward contexts, identifying key features associated with stabilization. This technical capture of temporal dependencies was verified by monitoring model convergence, ensuring that the stabilization trends observed in the expert scores were reflected in the model’s reduced training loss. This pattern is consistent with prior work on motor variability and optimal variability in rehabilitation and CP populations [17,18]. In the context of systematic reviews on motor learning in children with CP [17] and the theoretical notion of “optimal variability” as a key mechanism for adaptability and transfer [18], the observed trend toward stabilization in this study suggests that routine learning may reorganize athletes’ performance strategies into more efficient and consistent patterns. This supports the inclusion of longitudinal stability indicators as impact-relevant outcomes when evaluating AT-enabled interventions in severe CP.

For P3, although total scores remained low, the observed stabilization trend in later trials indicates that, in athletes with more severe impairments, structural changes in stability and variability may be more informative indicators of learning than absolute scores. By providing empirical accountability through the 4.69% reduction in trajectory variability, we address the criticism regarding post hoc rationalization of P3’s performance. Fatigue- and impairment-related constraints may limit observable short-term score gains even when movement strategies become more stable across repeated practice. The P3 case in this study illustrates that a multidimensional approach is essential for evaluating learning effects in highly impaired populations, reinforcing the need for long-term, individualized instructional plans (e.g., IEP-related planning in school contexts where applicable) that accommodate slower learning curves. This highlights the role of AI-based systems—validated by engineering metrics—as a sustainable diagnostic tool for long-term Individualized Education Program (IEP) planning. From an AT outcome/impact standpoint, this also implies that “meaningful change” should be operationalized with severity-responsive indicators (e.g., stabilization preceding score gains).

Taken together, these results show that: (1) depending on athlete skill level and task difficulty, the effects of routine learning may manifest as either score improvement or variability reduction; (2) the degree of organization of performance subcomponents differs across player–task combinations and can be observed in Cronbach’s α values and component score patterns; and (3) learning curves and time-series variability provide important indicators for explaining qualitative changes in Boccia skill acquisition. Crucially, the technical reliability established through an MSE of 1.14 and a 4.69% reduction in P3’s kinematic variance positions this AI-based routine-learning system as a validated measurement tool. This underscores that AI-based systems have potential not only as tools to enhance performance scores, but also as diagnostic instruments for monitoring changes in skill structure and progression through learning stages. Crucially, this positions the system as a measurement-oriented AT platform capable of documenting outcomes and impact in contexts where conventional evaluation is constrained by severity, rarity, and accessibility barriers.

### 4.2. Implications from the Perspective of Physical Education and EdTech

Ma et al. [27] reported that AI-based real-time feedback improved both movement quality and learning engagement in online physical education classes, while Ba et al. [28] showed that AI-assisted feedback, by analyzing performance data and contextual information, can foster self-regulated learning through personalized feedback. The solution used in this study, which models 29 kinematic variables using a regression-based AI approach integrated with a Bidirectional LSTM (Bi-LSTM) architecture, aligns with such EdTech research by providing individualized technical instruction. Critically, unlike purely descriptive EdTech tools, the reliability of this solution was empirically established through a validated MSE of 1.14 and a training loss reduction from 7.45 to 1.19, ensuring that the pedagogical feedback is grounded in technical rigor rather than subjective inference. In disability sport settings, this contributes to accessibility-oriented instruction by translating practice into interpretable, repeatable indicators that reflect the learner’s progression through the cognitive, associative, and autonomous stages.

First, AI-based routine-learning solutions can function as quantitative assessment tools that complement teachers’ subjective judgments. By visualizing changes in scores and time-series features over repeated practice, teachers and coaches can monitor performance changes in a data-driven way, providing a solid basis for adjusting training strategies and designing individualized feedback. Based on the observed trajectories, the system enables practitioners to detect “silent signals” of learning; for instance, in ‘stabilization-first’ patterns where mean scores stagnate, the detection of a 4.69% reduction in kinematic variability (as seen in P3) provides a quantitative justification for maintaining or adjusting coaching strategies that experts might otherwise overlook. For ‘plateau’ patterns (P1, P2), increasing task difficulty is recommended to challenge established routines. The consensus-based scoring procedure used in this study ensures that the AI’s automated regression outputs are grounded in high-level elite coaching standards, thereby bridging the gap between subjective expertise and objective data. The observed improvements in Distance and Pitch—indices directly linked to competitive performance—are consistent with the possibility that AI-based feedback was used to support practice refinement, although causal inferences cannot be drawn from the present design. In pediatric motor learning (including children with CP), feedback is more effective for retention when provided in an external focus format that emphasizes movement outcomes or environmental effects rather than the movement itself [33]. In this study, AI-generated information such as “predicted ball trajectory” and “deviation from target release velocity” exemplifies such external-focus feedback and may have supported self-regulated adjustment through outcome-referenced cues. By establishing an MSE of 1.14, this system functions as a highly reliable “digital coach,” providing the technical accountability required to move beyond anecdotal claims to evidence-based sustainable pedagogy.

Second, for student-athletes with disabilities, being able to directly view their own performance data has important educational significance Zhang et al. [32] emphasized that AI-based interventions for students with disabilities should be personalized, implying the need for individualized learner-specific baselines when interpreting performance data. The present study’s combined approach—comparing early and late phases by player–task and interpreting learning curves—accords with this principle. By visually confirming their validated learning curves, athletes can objectively recognize their transition from the high-variability cognitive stage to the more stabilized associative stage, supported by the knowledge that their progress is captured with a Mean Absolute Error (MAE) of 1.13. The process of visually confirming one’s own learning curve and score changes can move learners beyond passively receiving feedback, enabling them to recognize how they are changing and take a more active role in their learning. This is closely related to the development of self-understanding and self-regulation, key goals in inclusive and adapted physical education. From a technical standpoint, the markerless motion capture system used in this study has demonstrated accuracy and validity comparable to marker-based systems for analyzing upper-limb movements and postural control in children with CP [30,31]. This technical reliability, further evidenced by the model’s convergence (Loss: 7.45 → 1.19), provides a sustainable basis for cost-effective implementation in adapted PE settings.

Third, as Shieh et al. [22] evaluated the effects of assistive devices using AI-based kinematic analysis, the system employed in this study, with its ability to quantify movement refinement through 29 high-dimensional features, can be extended as an innovative evaluation platform for various assistive technologies and postural support devices. In future work, the detection of subtle changes—such as the 4.69% reduction in trajectory variance—can serve as a powerful metric for comparing learning curves between conditions with and without a particular support device. Schools and policymakers can evaluate whether the device produces meaningful functional benefits for students with disabilities, serving as a foundation for Individualized Education Program (IEP) planning. This directly connects AT adoption to validated longitudinal outcome/impact evidence, addressing the international research gap regarding objective monitoring in severe disability contexts.

### 4.3. Policy Implications

Boccia serves as a key disability sport that connects school-based physical education, community sport, and elite competition, providing crucial opportunities for sport participation among students with severe disabilities. Systematic reviews by Ferreira et al. [3,9] have pointed out that scientific research and evidence-based training models for Boccia remain limited. By demonstrating that AI-based routine-learning solutions can generate quantitative data and visualize the effects of repeated practice even for rare populations, this study suggests several preliminary policy implications for sustainable educational innovation. Critically, the establishment of technical accountability through a validated MSE of 1.14 and documented model convergence (Loss: 7.45→1.19) addresses the policy-relevant need for rigorous outcome documentation when adopting AT and accessibility-oriented infrastructures.

First, there is a need to integrate validated AI-based assessment and learning systems into adapted PE facilities and immersive sport environments such as VR/AR sports rooms. This would enable a shift from simple experiential content toward sustainable integrated platforms for assessment, training, and record management supported by empirical engineering metrics. The effects of sport-specific repeated training reported by Yahagi et al. [14] and Sufitriyono et al. [15], together with the learning curve and variability-reduction patterns observed in this study, provide quantitative evidence for answering practical questions such as “what type of training, and at what frequency, produces meaningful effects?” The implementation of the Bi-LSTM architecture to analyze the temporal structure of these patterns aligns with reviews suggesting that AI/ML approaches have the potential to play a central role in personalized management in CP [35] and has the potential to evolve into an intelligent decision-support system. The technical reliability confirmed in this study (MSE = 1.14) provides a foundation for scaling such systems into national-level inclusive excellence programs, ensuring that technology adoption is paired with longitudinally trackable, objective outcome indicators.

Second, accumulating and analyzing routine-learning data by functional level and classification grade could help refine sport-specific training guidelines. Zhang et al. [32] highlighted that the evidence base for AI-based interventions for students with disabilities remains limited, underscoring the need for disability- and learner-specific data when interpreting performance and progress. By documenting subtle progress—such as the 4.69% reduction in kinematic variance in the most severely impaired contexts—this study helps bridge this gap and serves as a foundational step for constructing national-level sustainable databases. Such databases can support accessibility-informed benchmarking and cross-setting outcome comparisons, providing policymakers with validated benchmarks for “meaningful change” that go beyond absolute performance scores.

Third, effective use of AI-based systems in practice requires a professional development framework that enhances adapted PE teachers’ and Boccia coaches’ capacity to use EdTech and interpret technical metrics. Training should go beyond basic device operation to encompass data interpretation, including an understanding of error metrics (MSE, MAE) and model stability (loss curves), ensuring that AI-driven instruction is ethically and technically grounded. This is essential for preventing the misinterpretation of outcome data—such as recognizing the AI-detected 4.69% reduction in kinematic variance as a meaningful indicator of the associative stage in severe CP—and ensuring that practitioners do not rely on “trust-based” claims but on validated evidence. Such comprehensive professional development is essential for ensuring that AI-based PE infrastructure becomes a sustainable educational practice rather than a one-off project to achieve long-term inclusive excellence.

### 4.4. Limitations and Future Directions

This study has clear limitations in terms of generalizability, as it involved a small number of Boccia athletes with disabilities and a limited set of routine tasks. With only three participants, the findings should be interpreted as exploratory, case-level evidence, particularly in light of the inter-individual variability and optimal variability emphasized in recent motor learning research [17,18]. However, given the extreme rarity of elite athletes with GMFCS level IV CP, the high-volume longitudinal data (694 trials) presented here provides a robust analysis that captures movement stabilization patterns often missed in larger cross-sectional studies. Moreover, all participants in this study had CP (two were adult elite athletes and one was a student-athlete), so the findings cannot be assumed to generalize to other impairment types, other Paralympic sports, or non-elite school populations. Future research should examine the effects of AI-based routine learning in larger, multi-site studies that include diverse ages, classification levels, and genders to ensure the educational sustainability of these findings. This is particularly important for enhancing model robustness in rare disease contexts, where high intra-subject variability and limited datasets necessitate advanced data balancing and interpretation strategies [37]. In this context, the scientific contribution of our work lies in the transferability of the measurement framework rather than population-level generalization, positioning this AI-enabled approach as a replicable model for objective outcome monitoring in other rare and severe disability contexts. In addition, the effect sizes reported here are based on simplified calculations using repeated-measures data and should be viewed as indices of practical change magnitude rather than strictly inferential statistics. While we have addressed technical rigor in this study by reporting standardized engineering metrics—specifically a validated Mean Squared Error (MSE) of 1.14 and a Mean Absolute Error (MAE) of 1.13—further external validation on independent cohorts remains necessary to evaluate out-of-sample robustness beyond the current case-series dataset.

From a design perspective, another limitation is that the present study used a single-group repeated-measures design. Employing quasi-experimental designs such as condition-comparison studies (e.g., Tsai et al. [11]), would allow for clearer identification of the relative advantages of AI-driven innovation compared with traditional coaching. For example, a design comparing “AI feedback + coach instruction” versus “coach instruction only” would provide more robust evidence regarding added value for long-term pedagogical sustainability than the current exploratory case-series design can offer. Furthermore, while the current analysis utilized descriptive summaries and effect sizes, future work should strengthen the within-athlete analytical framework by adding established single-case statistical methods, such as Tau-U, randomization tests, or other effect estimation methods suitable for time-series data, to quantify the magnitude and consistency of changes across repeated trials beyond visual inspection. Notably, the empirical evidence of a 4.69% reduction in kinematic variance for Player 3 (*p* > 0.05, descriptive trend) provides a useful starting point for such future statistical modeling, distinguishing technology-enabled benefits from standard practice effects in stagnant performance contexts.

Regarding the AI model, this study has moved beyond a high-level description by providing a systematic evaluation of its predictive accuracy and training convergence within the current report. We have explicitly reported the model’s loss reduction (from 7.45 at epoch 1 to 1.19 at epoch 21) and LOSO cross-validation results to establish empirical accountability. Importantly, a clear distinction must be made between the behavioral improvement of the athletes and the technical optimization of the AI model. While this study detailed the technical loss reduction and gradient optimization of the Bi-LSTM model, future work should continue to refine these parameters by separating dedicated test sets for performance evaluation and comparing alternative time-series modeling approaches. In addition, given emerging evidence that markerless motion capture accuracy can vary depending on movement patterns and severity of impairment in children with CP [30,31], future studies should explicitly report model performance and measurement error characteristics in these highly specific populations. Moreover, the current analyses were based primarily on consensus-based expert ratings; while we have reported the technical alignment between these ratings and the AI-generated scores (MSE = 1.14), additional agreement and calibration analyses (e.g., correlation coefficients, ICC, or Bland–Altman plots) could further strengthen diagnostic interpretation in future studies, rather than representing a prerequisite that is absent from the current validation evidence.

Finally, although the original IRB protocol included a mixed-methods design with interviews and observations, actual data collection focused on quantitative performance data. As a result, the study had limited ability to capture in depth the experiences and perceptions of athletes, teachers, and caregivers. In line with Ma et al. [27] and Ba et al. [28] future research should adopt mixed-methods designs that integrate technology acceptance (TAM), teacher knowledge structures (TPACK), and school context in order to explore how AI-based routine-learning solutions are accepted in practice and under what conditions they become sustainable. In disability sports, ethical considerations around user autonomy, privacy, and data-driven instruction are essential when introducing AI technologies. Therefore, future work should be complemented by qualitative studies that examine ethical implementation strategies and user experiences related to AI-based sustainable systems. This will help clarify accessibility as both a measurable outcome and a lived experience (e.g., perceived usefulness, burden reduction, participation facilitation) alongside performance indicators.

## 5. Conclusions

This study conducted a longitudinal case series that quantitatively examined changes in performance during repeated practice when an innovative AI-based routine-learning solution, utilizing a Bidirectional Long Short-Term Memory (Bi-LSTM) architecture to model 29 specific kinematic features, was applied to a rare population of Boccia athletes with CP. To establish the system’s role as a credible measurement tool, the AI model was technically validated through Leave-One-Subject-Out (LOSO) cross-validation, yielding a Mean Squared Error (MSE) of 1.14 and a Mean Absolute Error (MAE) of 1.13. Across three players and seven player–task combinations, utilizing a high-volume dataset of 694 trials, mean total scores improved in most routines, with particularly pronounced gains observed in several high-difficulty tasks. These learning curves effectively documented the athletes’ progression from the high-variability cognitive stage to more stabilized associative and autonomous stages. The regression-based AI approach demonstrated its technical sensitivity by detecting subtle movement refinements—specifically a 4.69% reduction in joint trajectory variability for the most severely impaired athlete (P3)—even in the absence of mean-score increases. In such instances, stabilization and reduced fluctuation emerged as empirically substantiated indicators of progress within the cognitive stage, suggesting that multidimensional outcome reporting is necessary for disability-focused evaluation.

Taken together, these findings suggest that an AI-based routine-learning solution may serve as a feasible and sustainable pedagogical tool for individualized instruction and data-driven feedback in disability sports. The technical credibility of the system was reinforced by the documentation of model convergence, where training loss was reduced from 7.45 to 1.19, ensuring that the predictive metrics align with expert consensus ratings. In particular, player–task–specific learning curves and variability indices can be used as innovative diagnostic information that goes beyond simple score changes to indicate which components stabilize through what trajectories. This provides a vital foundation for the long-term sustainability of Individualized Education Programs (IEPs) in school-linked settings by offering objective evidence of progress that traditional observational methods might overlook. Aligned with an outcome-oriented AT perspective, the system can also be conceptualized as a validated measurement-and-feedback platform that supports accessible monitoring in rare and severe disability populations.

When linked with adapted physical education and disability sport policy, the system proposed in this study has the potential to evolve into an infrastructure that supports increased sport participation, talent development, and the promotion of health. The establishment of technical accountability in this report—moving beyond trust-based claims to report standardized engineering metrics—strengthens the case for adopting AI-enabled motion analysis in elite disability sport settings. Future evidence building should emphasize outcome/impact frameworks that are disability-responsive (e.g., detecting kinematic stabilization) and usable for assistive-technology adoption decisions in schools and training systems.

Future research should extend this work by including more diverse participants and appropriate comparison groups within quasi-experimental designs. While this study has established initial measurement validity through agreement analysis with expert ratings and the reporting of MSE (1.14), future work should further explore statistical correlation using intraclass correlation coefficients to ensure continued technical rigor. Through these efforts, the educational and policy value—and the limitations—of AI-driven innovations can be clarified more systematically and translated into sustainable practices in schools and disability sport settings to bridge the gap between subjective coaching and evidence-based sustainable pedagogy. In particular, documenting the accessibility-related impacts of modeling 29 high-dimensional kinematic features will strengthen the contribution of this line of work to assistive technology and disability outcome research.

## Figures and Tables

**Table 1:** General characteristics and classification of the participating Boccia players.

Player ID	Sex	Age (Years)	Diagnosis (Type of CP)	GMFCS Level	Bisfed Boccia Class
P1	Male	24	Spastic quadriplegia	IV	BC2
P2	Male	31	Spastic quadriplegia	IV	BC3
P3	Male	16	Spastic quadriplegia	IV	BC1

**Table 2:** Overview of routine-learning performance by player–task combination (total score).

Player-Task	Number of Trials	Mean Total Score	SD of Total Score
P1-T1	33	7.19	0.14
P1-T2	110	8.79	0.12
P1-T3	110	7.46	0.49
P2-T2	110	9.17	0.37
P2-T3	110	8.17	1.04
P3-T2	111	4.29	0.15
P3-T3	110	3.99	0.14

**Table 3:** Comparison of total scores between the first 10 and last 10 trials.

Player-Task	Early Phase Mean	Early Phase SD	Late Phase Mean	Late Phase SD	Change (Late − Early)	Cohen’s *d*
P1-T1	7.04	0.12	7.20	0.07	0.16	1.63
P1-T2	8.71	0.10	8.80	0.18	0.09	0.62
P1-T3	6.93	0.12	8.38	0.40	1.45	4.91
P2-T2	9.22	0.15	9.21	0.17	−0.01	−0.06
P2-T3	6.44	0.13	8.80	0.52	2.36	6.23
P3-T2	4.29	0.13	4.21	0.14	−0.08	−0.59
P3-T3	4.00	0.18	4.02	0.11	0.02	0.13

**Table 4:** Mean scores for performance subcomponents by player–task (0–10 scale).

Player-Task	Postural	Seated	Hand	Arm	Release	Distance	Pitch	Total Score
P1-T1	7.09	7.15	6.97	6.94	6.48	7.75	6.69	7.19
P1-T2	9.05	9.04	9.31	9.12	9.31	6.96	8.01	8.79
P1-T3	8.68	8.10	6.66	6.49	6.71	7.72	7.65	7.46
P2-T2	9.85	9.72	9.51	9.23	8.95	8.00	8.99	9.17
P2-T3	8.09	8.32	8.28	7.79	7.62	8.76	8.43	8.17
P3-T2	4.60	4.58	4.84	4.40	4.13	3.31	4.16	4.29
P3-T3	4.86	4.87	3.92	3.47	3.17	3.51	4.13	3.99

**Table 5:** Internal consistency (Cronbach’s α) of the performance rating scale by player–task.

Player-Task	Cronbach’s α
P1-T1	0.68
P1-T2	0.47
P1-T3	0.93
P2-T2	0.96
P2-T3	0.99
P3-T2	0.49
P3-T3	0.65

## Data Availability

The data presented in this study are available upon request from the corresponding author. The data were not publicly available because of the protection of personal information.
